# Hacking Pancreatic Cancer: Present and Future of Personalized Medicine

**DOI:** 10.3390/ph14070677

**Published:** 2021-07-15

**Authors:** Alessandro Di Federico, Valentina Tateo, Claudia Parisi, Francesca Formica, Riccardo Carloni, Giorgio Frega, Alessandro Rizzo, Dalia Ricci, Mariacristina Di Marco, Andrea Palloni, Giovanni Brandi

**Affiliations:** 1 Division of Medical Oncology, IRCCS Azienda Ospedaliero-Universitaria di Bologna, 40138 Bologna, Italy; alessandrodifederico1@gmail.com (A.D.F.); valentina.t91@gmail.com (V.T.); parisiclaudia168@gmail.com (C.P.); formica.francesca@gmail.com (F.F.); riccardo.carloni2@studio.unibo.it (R.C.); giorgio.frega2@unibo.it (G.F.); rizzo.alessandro179@gmail.com (A.R.); dalia.ricci@gmail.com (D.R.); mariacristina.dimarco@unibo.it (M.D.M.); giovanni.brandi@unibo.it (G.B.); 2 Department of Specialized, Experimental and Diagnostic Medicine, University of Bologna, Via Giuseppe Massarenti, 9, 40138 Bologna, Italy

**Keywords:** pancreatic cancer, immunotherapy, targeted therapy, tyrosine kinase inhibitor, personalized medicine, metabolism, tumor microenvironment, PARP inhibitors, genomic, DNA damage

## Abstract

Pancreatic cancer (PC) is a recalcitrant disease characterized by high incidence and poor prognosis. The extremely complex genomic landscape of PC has a deep influence on cultivating a tumor microenvironment, resulting in the promotion of tumor growth, drug resistance, and immune escape mechanisms. Despite outstanding progress in personalized medicine achieved for many types of cancer, chemotherapy still represents the mainstay of treatment for PC. Olaparib was the first agent to demonstrate a significant benefit in a biomarker-selected population, opening the doors for a personalized approach. Despite the failure of a large number of studies testing targeted agents or immunotherapy to demonstrate benefits over standard chemotherapy regimens, some interesting agents, alone or in combination with other drugs, have achieved promising results. A wide spectrum of therapeutic strategies, including immune-checkpoint inhibitors tyrosine kinase inhibitors and agents targeting metabolic pathways or the tumor microenvironment, is currently under investigation. In this review, we aim to provide a comprehensive overview of the current landscape and future directions of personalized medicine for patients affected by PC.

## 1. Introduction

Pancreatic cancer (PC) is an aggressive disease, accounting for around 3% of all new cancer diagnoses in the United States of America and Europe [1]. Up to 80% of patients have a locally advanced or metastatic disease at the time of presentation and a poor prognosis, with an overall life expectancy of 5 years at 4–9% [2,3].

As for other recalcitrant tumor types, advances in the identification of clinically relevant alterations will be remarkable in redefining therapeutic strategies toward a personalized treatment. Real-world data have suggested that adopting precision medicine in PC patients with actionable alterations, defined as molecular alterations for which there is clinical or strong preclinical evidence of a predictive benefit from a specific therapy, can have a great impact on patients’ outcomes [4,5]. A retrospective analysis of the “Know Your Tumor” trial demonstrated a significant overall survival (OS) improvement of 1 year among patients harboring actionable molecular alterations (accounting for 25% of total PC patients) who received matched therapy compared to those who did not [6].

Recent approvals of tumor-agnostic agents, such as pembrolizumab for microsatellite instability-high (MSI-H) tumors and NTRK inhibitors for NTRK fusion-positive tumors, have provided two novel therapeutic possibilities for selected PC patients [7,8,9,10]. Unfortunately, due to the rarity of these alterations, the proportion of patients who may benefit from these treatments is limited. Further investigations in preclinical and clinical research are mandatory, considering the complex genomic landscape of PC and the urgent need of therapeutic improvements. In this work, we performed an extensive review of the literature to provide a comprehensive and timely overview of the current landscape and future perspectives of personalized medicine in PC.

## 2. Biomolecular Landscape of Pancreatic Cancer

Based on gene expression, PC has been clustered into subtypes with different biological and prognostic features. A large genomic characterization of PC reviewed the three principal classifications published in previous years, in light of new considerations concerning neoplastic cellularity and tumor purity and new techniques of deep genomic, transcriptomics, and proteomics [11]. Based on gene expression, high purity samples can be classified as basal-like or squamous and classical or pancreatic progenitor, while low purity samples can be classified as aberrantly differentiated endocrine exocrine (ADEX), exocrine-like, immunogenic, and quasimesenchymal, most likely due to gene expression from stroma cells [11].

High-frequency gene mutations in PC involve KRAS (93%), TP53 (72%), CDKN2A (30%), SMAD4 (32%), RNF43 (7%), ARID1A (6%), TGFβR2 (5%), GNAS (8%), RREB1 (5%), and PBRM1 (4%), but other potentially druggable mutations with a lower prevalence were revealed using combined analysis [11]. Amplification of GATA6, ERBB2, KRAS, AKT, and MYC, and deletion of CDKN2A, SMAD4, ARID1A, and PTEN were the most frequently identified somatic copy number aberrations. Germline mutations of known predisposition genes, such as BRCA1/2, PALB2, TP53, CDKN2A, ATM, PRSS1, STK11, MLH1, MSH2, MSH6, PMS2, and EPCAM, were observed in 8% of patients, with a significant enrichment in the KRAS wild-type tumors [11,12]. These tumors frequently harbored mutations in the RAS-MAPK pathway, leading to its hyperactivation, including in absence of the KRAS mutation. Moreover, protein expression profiling in these samples revealed a high activity of the mTOR pathway. Analyses of DNA methylation, combined with mRNA expression data, showed the hypermethylation of several genes, especially tumor suppressor genes or genes coding for regulator miRNAs, with consequent epigenetic silencing [11].

Currently, and considering the available knowledge on PC biology and targeted therapies, almost 25% of PC patients may be a candidate for precision medicine [13]. Many genomic alterations are targetable with specific drugs, but most clinical trials have failed to demonstrate practice-changing results. However, it is essential to perseverate with molecular research to improve the outcome of PC patients.

## 3. DNA Damage Response and Repair Genes (DDR)

DNA damage response and repair mechanisms are necessary to maintain the integrity of cell DNA. The dysfunction of DNA repair machinery ultimately leads to the accumulation of somatic mutations, increasing the risk of developing cancer [14]. Six main DNA repair pathways operate depending on the type of DNA damage. Single-stranded breaks (SSBs) can lead to the activation of four different repair mechanisms based on the damage: base excision repair, nucleotide excision repair, mismatch repair, and translesion synthesis. Damage involving both DNA strands (double-stranded breaks) can activate two compensatory pathways: homologous recombination repair (HRR), an accurate system that uses a complementary strand from a sister chromatid to reproduce the original DNA sequence, and non-homologous end-joining, which is more error-prone as it utilizes no or limited homologous sequences to restore the damaged strand [15]. An increasing number of genes that help the DNA repair machinery to function have been identified, and the inherited deficiency of several genes, such as BRCA1/2, ATM, and PALB2, are linked to the predisposition of developing PC [16,17,18]. In particular, germline or somatic mutations of BRCA1, BRCA2, PALB2, ATM, and CHEK2 have been reported in 20% of PDAC, while a deficiency of HRR genes was documented in 15.4% of PC and ATM mutations in 9–18% of PC [19,20,21]. DDR deficiency is known to co-segregate with improved response to platinum derivatives consistently with the mechanism of action of these agents. Moreover, deficient DDR machinery can enhance an immune response in multiple ways, providing the rationale for the combination of DDR-targeting agents and immunotherapy [22]. The number of antitumor agents taking advantage of DDR deficiency is constantly expanding and many trials are ongoing (Table 1), hopefully to provide increasing treatment possibilities for PC patients.

### 3.1. PARP Inhibitors

**Figure 1:**
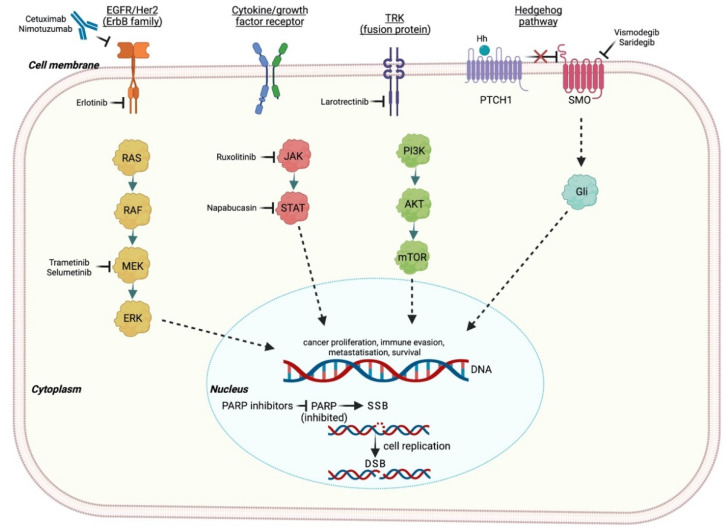
Main transmembrane receptor and intracellular pathways evaluated as potential therapeutic targets in PC. EGFR: epidermal growth factor receptor; HER-2: human epidermal growth factor receptor 2; NTRK: neurotrophic tyrosine receptor kinase; PTCH1: 12-transmembrane patched protein 1; SMO: 7-transmembrane smoothened protein: RAS: rat sarcoma; RAF: rapidly accelerated fibrosarcoma; MEK: mitogen-activated protein kinase; ERK: extracellular signal-regulated kinase; JAK: Janus kinase; STAT: signal transducer and activator of transcription; PI3K: phosphoinositide-3-kinase; mTOR: mechanistic target of rapamycin; Gli: 5-zinc-finger transcription factor; SSB: single-stranded break; DSB: double-stranded break; PARP: poly ADP-ribose polymerase. Created with BioRender.com (accessed on 14 July 2021).

Poly-ADP-ribose polymerase inhibitors (PARPis) take advantage of the concept of synthetic lethality, which is defined by the combination of inactivating mutations in two or more different genes essential for cell integrity, inducing cell death [23]. The PARP-1 gene is involved in several phases of the DNA repair machinery, especially in preventing SSBs [24]. Thus, PARPis determine the accumulation of unrepaired SSBs, which are converted into DSBs during cell replication (Figure 1). In this scenario, the concomitant presence of HRR deficiency, such as BRCA1/2 inactivating mutations, enhances a predisposition to synthetic lethality [25].

Following this, we describe the current knowledge and the incoming opportunities concerning PARP inhibition in PC.

#### 3.1.1. Olaparib

At the time of this review, olaparib represents the only approved PARPi in PC. The phase II trial conducted by Kaufman et al. evaluated olaparib monotherapy (400 mg twice daily) in patients with recurrent cancer that harbor germline BRCA1/2 mutations [26]. Twenty-three patients with PC who received a prior treatment with gemcitabine were included in the study. The objective response rate (ORR) was 21.7% (95% CI, 7.5 to 43.7), and stable disease (SD) lasting ≥8 weeks was observed in 34.8% of patients. A promising median progression-free survival (PFS) of 4.6 months and overall survival (OS) of 9.8 months were also documented. Olaparib approval in PC was obtained following the results of the randomized, double-blind, placebo-controlled, phase III POLO trial. A total of 154 patients with metastatic PC and a germline mutation of BRCA1/2 who did not progress during first-line platinum-based chemotherapy were randomized to receive olaparib monotherapy (300 mg twice daily) or placebo as maintenance therapy [27]. Median PFS was significantly prolonged in the olaparib group (7.4 vs. 3.8 months; HR 0.53; CI 95% 0.35–0.82; *p* = 0.004), while median OS was similar between groups. Notably, two studies evaluating the combination of olaparib and chemotherapy are discontinued due to severe toxicity issues [28,29].

#### 3.1.2. Veliparib

Veliparib is an oral PARPi with lower PARP trapping ability compared with Olaparib [30,31]. A single arm, phase II trial tested this agent at a dose of 400 mg twice daily in previously treated stage III/IV PC patients with a germline BRCA1/2 or PALB2 mutation [32]. Unfortunately, no tumor response was documented, but SD lasting ≥4 months was observed in 25% of patients. The addition of veliparib (80 mg twice daily on days 1 to 12 every 3 weeks) to cisplatin, plus gemcitabine chemotherapy (25 mg/m^2^ and 600 mg/m^2^, respectively, both on days 3 and 10), was recently evaluated in a two-arm, phase II trial enrolling 50 patients with stage III/IV untreated PC and a germline BRCA1/2 or PALB2 mutation [33]. Both arms demonstrated high antitumor activity, with a disease control rate (DCR) of 100% in the combination arm versus 78.3% in the chemotherapy alone arm (*p* = 0.02), although no significant difference in ORR was observed (74.1% vs. 65.2% in the triplet and doublet arm, respectively; *p* = 0.055). Conversely, a notable increase of hematologic toxicities was reported in the combination arm. Veliparib was also tested, in addition to modified FOLFIRI, as a second-line treatment for metastatic PC patients, showing no additional benefit among biomarker unselected patients [34].

#### 3.1.3. Rucaparib

The single-arm phase II RUCAPANC study investigated efficacy and safety of the oral PARPi rucaparib (600 mg twice daily) among 19 patients with pretreated locally advanced/metastatic PC harboring a germline or somatic BRCA1/2 mutation [35]. Rucaparib demonstrated an acceptable toxicity profile and provided clinical benefits, as ORR was 15.8% and DCR was 31.6%. Rucaparib (600 mg twice daily) was then investigated as maintenance monotherapy in a single-arm phase II trial, enrolling patients with advanced pancreatic cancer and harboring a BRCA1/2 or PALB2 mutation but who did not show PD following at least 4 months of platinum-based chemotherapy [36]. Again, this agent showed encouraging antitumor activity with a safe toxicity profile, as median PFS was 9.1 months, ORR was 36.8%, and 89.5% of patients achieved DCR lasting ≥8 weeks.

#### 3.1.4. Talazoparib

Talazoparib is a novel, potent oral PARPi. The two-part, dose-escalation, phase I trial by de Bono et al. tested this agent in patients with advanced solid tumors and a germline BRCA1/2 mutation [37]. Talazoparib showed a tolerable profile and promising antitumor activity, as ORR was 20% among 20 patients with advanced PC.

Ongoing trials investigating the use of PARPis alone or combined with different agents for the treatment of advanced PC patients are summarized in Table 1. These agents have demonstrated a synergistic effect with programmed death-1/programmed death-ligand 1 (PD-1/PD-L1) inhibitors in murine models, possibly due to PD-L1 upregulation in the tumor microenvironment [38,39]. Moreover, the addition of a MEK inhibitor to a PARPi and a PD-(L)1 inhibitor is currently under evaluation to overcome RAS-mediated resistance to PARPis in RAS-mutant PC, which was reported in preclinical models [40].

### 3.2. Further DDR Targeting Agents

PARPis represent only a small part of the wide scenario of DDR gene inhibition. The interest in DDR genes has risen in recent years as an increasing number of specific agents targeting these pathways is in the testing or development phase. The ataxia-telangiectasia mutated (ATM) gene plays a central role in DNA damage response and DSB repair, and its germline mutation is known to produce a specific syndrome, as well as increasing the risk of developing several types of cancer, including PC [41]. Once the ATM signal is disrupted, the cell relies on downstream ATR and CHK1/2 pathways to ensure DNA repair, arresting the cell cycle and preventing the DNA fork from collapsing. These functions make them two possible targets in ATM-deficient tumors, which account for 9–18% of sporadic PC [42,43]. Consistently, a sensitization of ATM-mutated PC cell lines to PARP, ATR, and CHK1/2 inhibitors, alone or in combination with other agents, have been demonstrated in preclinical studies [44,45,46]. Considering these data, several ongoing clinical trials are evaluating the potential of ATM, ATR, and CHK1 inhibitors in patients with PDAC (Table 1).

WEE1 regulates the G2 DNA damage checkpoint in concert with CHK1 during cell replication, which delays the completion of mitosis of cells that suffer genomic damage, thus, increasing cell viability [47]. These functions provide the rationale of blocking WEE1 to increase the efficacy of DNA damaging agents and to enhance synthetic lethality. MK-1775 (also known as AZD1775 and adavosertib), a WEE1 inhibitor, has been tested in combination with several DNA damaging agents in preclinical studies, such as gemcitabine, mitomycin C, and platinum derivatives [48,49]. A phase I trial enrolling 176 patients with refractory solid tumors evaluated adavosertib in combination with chemotherapy (either carboplatin, cisplatin, or gemcitabine) and documenting a 10% ORR. The response rate was significantly higher (21%) in the subpopulation of TP53-deficient patients as these tumors strongly depend on WEE1 activation to arrest the cell cycle in response to DNA damage [50,51]. Recently, a dose-escalation trial enrolling treatment-naïve, locally advanced PC patients documented a median OS of 21.7 months (90% CI, 16.7 to 24.8 months), and a median PFS of 9.4 months (90% CI, 8.0 to 9.9 months) with a combination of adavosertib, gemcitabine, and radiation therapy (RT) [52]. A phase I/II trial evaluating the addition of adavosertib to chemotherapy in metastatic PC is ongoing (Table 1).

## 4. MEK Inhibitors

The RAS/RAF/MEK/ERK mitogen-activated protein kinase (MAPK) cascade is deeply involved in the pathogenesis of PC, especially in tumors harboring RAS or BRAF mutations [53]. Trametinib, an oral and selective tyrosine kinase inhibitor of mitogen/extracellular signal-related kinase (MEK) 1/2, showed modest antitumor activity as monotherapy in PC in a phase I trial [54]. Following this, its combination with gemcitabine was compared with gemcitabine monotherapy in a randomized phase II trial enrolling previously untreated metastatic PC. However, no differences in terms of median OS, PFS, and ORR between the two arms were documented, including the KRAS-mutant subpopulation [55]. Similar disappointing results were obtained with pimasertib, another MEK1/2 inhibitor [56]. Among the possible mechanisms of resistance lies the hyperactivation of the epidermal growth factor receptor (EGFR), which prevents apoptosis through a feedback activation of the AKT-PI3K pathway [57]. This concept produced the idea of performing a double-blockade on MEK and EGFR. A single-arm phase II trial tested the combination of erlotinib, an EGFR inhibitor, and selumetinib, an MEK inhibitor, among 46 pretreated, advanced PC patients [58]. Low antitumor activity was documented by the proportion of patients who had SD lasting ≥ 6 weeks (41%) and decreased levels of carcinoembryonic antigen (CEA) (38%). However, no objective response was documented. Following the failure of this trial, the double-blockade strategy was focused on a target downstream of MEK and AKT in a phase II trial testing selumetinib with MK-2206 (a selective pan-AKT inhibitor) or modified-FOLFOX (oxaliplatin, 85 mg/m^2^ intravenous, and fluorouracil, 2400 mg/m^2^ intravenous infusion over 46–48 h) in patients with metastatic, chemotherapy-refractory PC [59]. Again, the combination treatment did not show the expected efficacy, as median OS and PFS were both shorter in the experimental arm as compared with the control arm.

## 5. EGFR Inhibitors

EGFR is a transmembrane tyrosine kinase receptor in which aberration leads to cell growth, proliferation, and survival. EGFR overexpression occurs frequently in PC, correlating with advanced disease [60]. The mutation status of the EGFR tyrosine kinase domain is a predictive factor for therapy with EGFR inhibitors in non-small cell lung cancer, but the same status applied to PC remains unclear. The association between gemcitabine and erlotinib, an EGFR inhibitor, and placebo was tested among 569 patients with advanced PC in a phase III trial [61]. Median OS results were slightly but significantly prolonged among patients in the erlotinib plus gemcitabine arm (6.24 months vs. 5.91 months; HR 0.82; CI 95% 0.69–0.99; *p* = 0.038), and median PFS was improved in the combination arm (3.75 months versus 3.55 months; HR 0.77; CI 95% 0.64–0.92; *p* = 0.004).

Investigators performed a molecular subset analysis, including 26% of enrolled patients, in which they analyzed the EGFR gene copy number and KRAS mutation status; nevertheless, they were not a predictive marker of survival benefit from the association of erlotinib with gemcitabine [62]. Aiming to explore the effects of erlotinib on subsequent treatments, the phase III LAP07 trial enrolled 449 patients with advanced PC to receive chemotherapy or chemoradiotherapy following a prior randomized phase in which they were treated with either gemcitabine alone or in combination with erlotinib [63]. Results were disappointing, as no difference was documented between the arms, leading to trial discontinuation for futility at the interim analysis. A subsequent trial analyzed the efficacy of the combination between gemcitabine and erlotinib versus gemcitabine alone depending on the presence of EGFR mutations among 88 chemotherapy-naïve metastatic PC patients [64]. The activating mutation in EGFR involved exon 20 (50%), exon 19 (37%), exon 21 (10%), and exon 18 (3%). Improved outcomes were documented with the addition of erlotinib to gemcitabine, either in terms of median PFS (3.8 months vs. 2.4 months; *p* < 0.001) or OS (7.2 months vs. 4.4 months; *p* < 0.001). Predictably, patients harboring EGFR mutations received the greatest benefit from the combination strategy, with longer median PFS (5.9 months vs. 2.4 months; *p* = 0.004) and OS (8.7 months vs. 6.0 months; *p* = 0.044) compared with EGFR wild-type patients. The most common mutation was L778P in exon 20, representing 24% of all mutations. Patients harboring the L778P mutations achieved improved DCR with the addition of erlotinib to gemcitabine as compared with gemcitabine alone. Other mutation sites failed to demonstrate a significant difference in response and DCR [64].

Using monoclonal antibodies, the transmembrane EGFR receptor can be targeted from outside the cell. One of the first agents created for this purpose is cetuximab, an antagonist chimeric monoclonal antibody with high affinity and specificity for EGFR [65]. A recent meta-analysis evaluated four randomized controlled trials testing cetuximab in patients with advanced PC, documenting no difference between the cetuximab and non-cetuximab groups, either in terms of OS (HR 1.04; CI 95% 0.90–1.19; *p* = 0.60), PFS (HR 1.06; CI 95% 0.93–1.22; *p* = 0.36), or ORR (Odds Ratio 0.99; CI 95% 0.66–1.49; *p* < 0.96) [66].

The association of nimotuzumab, another EGFR-targeting humanized monoclonal antibody, and gemcitabine was tested as a first-line treatment in 18 patients with advanced PC [67]. ORR was 11.1% and DCR was 55.6%. Median PFS and OS were 3.71 months and 9.29 months, respectively. More recently, the association of nimotuzumab and gemcitabine as a first-line treatment improved the 1-year OS rate (3.8% vs. 15.8%; HR 0.32; 95% CI, 0.13–0.84) in a double-arm, phase IIb trial, conducted on 186 patients with advanced, KRAS wild-type PC [68].

## 6. KRAS Targeting Agents

Kirsten rat sarcoma viral oncogene homolog (KRAS), an isoform of the RAS family proteins, is a small cytoplasmic protein with GTPase activity. KRAS represents the most frequent genomic alteration in PC, accounting for over 90% of cases. Activating mutations of this oncogene results in the promotion of cell growth, proliferation and survival, and have an initiating role in carcinogenesis [69]. A total of 98% of KRAS mutations occur in exon 2 (codon 12), with G12D and G12V being the most common mutations, followed by G12A/C/S (2% each), and G12L/F (<1%). Less frequently, mutations have been observed to involve codon 13 (<1%) and codon 61 (<1%) [70]. Due to its prevalence, targeting KRAS or its downstream pathway may be crucial in PC. Nevertheless, developing effective KRAS inhibitors is one of the most challenging objectives in oncologic research. Despite this, several KRAS-binding small molecules (e.g., Sotorasib, Adagrasib) have been recently developed to irreversibly inhibit the G12C missense mutant of KRAS, showing encouraging results in various cancer types, including non-small cell lung cancer. These agents might thus represent a promising strategy in the small proportion of PC harboring the G12C mutation [71]. Farnesylation is essential for the membrane anchorage of RAS proteins and the consequent RAS activity. Farnesyltransferase inhibitors competitively inhibit farnesyl protein-transferase, the enzyme responsible for farnesylation. The addition of farnesyltransferase inhibitor tipifarnib to gemcitabine in a phase III trial failed to improve the outcomes compared with placebo. Nonetheless, the preclinical and clinical activity demonstrated by salisarib, a farnesylcysteine mimetic that selectively disrupts the association of active RAS proteins with the plasmatic membrane, warranted the need for further investigation in phase II trials [72,73,74]. To date, multiple strategies aiming to target KRAS-mutant PC are under evaluation through ongoing clinical trials (Table 2).

## 7. Targeting Tumor Microenvironment: The First Yet Harder Obstacle to Overcome

PC has a peculiar tumor microenvironment which contributes to its resistance to different kinds of treatments. It is characterized by the presence of dense and heterogeneous stroma, mainly composed of cells with different functions, such as fibroblasts, myofibroblasts, immune cells and pancreatic stellate cells, blood vessels, extracellular matrix, and soluble proteins [75]. Together, these components prompt tumor growth and metastatic spread, and exert a high hydrostatic pressure within tumor vessels, which may limit cell trafficking, particularly of immune cells [76]. Targeting the tumor microenvironment may therefore be a valid strategy to overcome drug resistance and to favor immune infiltration in PC.

### Hedgehog Pathway

Hedgehog (Hh) signaling is an essential pathway during embryogenesis [77]. It is composed of Sonic Hh (SHh), Indian Hh, and Desert Hh, 12-transmembrane patched proteins (PTCH 1 and PTCH 2), 7-transmembrane smoothened protein (SMO), and 5-zinc-finger transcription factors (Gli1, Gli2, and Gli3) [78]. PTCH actively suppresses the pathway by inhibiting SMO; nevertheless, the binding of SHh, a secreted Hh ligand which is abnormally expressed in over 70% of PC, to PTCH prevents SMO inhibition, leading to the activation of the pathway [79]. The Hh pathway has an early and critical role in the genesis of PC. The maintenance of Hh signaling favors aberrant proliferation, while SHh is fundamental for the maintenance of cancer stem cells [79,80]. In fact, SHh activates pancreatic stellate cells, promoting a desmoplasia and hypoxic microenvironment by producing cytokines, chemotactic factors, growth factors, and an excessive extracellular matrix [81,82]. The resulting microenvironment favors neoplastic initiation and development, as well as tumor invasion, metastasization, immune escaping, and treatment resistance [81].

Vismodegib, an SMO antagonist, was evaluated in combination with gemcitabine in a phase Ib/II trial, enrolling 106 previously untreated PC patients, yet failing to improve the outcomes of the chemotherapeutic agent compared with placebo [83].

Another SMO antagonist, saridegib, was tested in combination with 5-fluorouracil, leucovorin, irinotecan, and oxaliplatin (FOLFIRINOX) in a phase I trial, and in addition to gemcitabine in a phase II trial, both conducted on previously untreated, advanced PC patients [84,85]. Despite early evidence of antitumor activity and acceptable safety reported by the phase I trial, the combination with gemcitabine evaluated in the phase II study showed a detrimental effect of the experimental agent, leading to the discontinuation of the trial after a preliminary analysis.

## 8. Microenvironment Targeting Agents

Specific agents have been studied in preclinical and clinical PC models aiming to enhance drug delivery. Pegvorhyaluronidase alfa (PEGPH20) demonstrated to degrade hyaluronic acid in the TME in preclinical studies, thereby increasing drug delivery to cancer cells. PEGPH20 was investigated in a phase II trial enrolling 279 previously untreated PC patients, randomly assigned to nab-paclitaxel and gemcitabine with or without PEGPH20 [86]. Median PFS was longer in the experimental arm (HR: 0.73; 95% CI, 0.53–1.00; *p* = 0.049), with superior benefit in patients whose tumor had high levels of HA. ORR was 45% versus 31%, but no OS benefit was documented.

PEGPH20 was also tested in combination with FOLFIRINOX in a phase Ib/II trial, enrolling patients with metastatic PC; nevertheless, its addition resulted to be detrimental in terms of both toxicity and survival [87].

TGF-β represents another potential target in PC, as its dysregulation is capable in promoting cell growth, epithelial-to-mesenchymal transition, extracellular matrix remodeling, and immunosuppression [88]. Galunisertib, a type I TGF-β receptor inhibitor, was investigated in combination with gemcitabine in a phase Ib/II trial as a first-line treatment for advanced PC [89]. A number of 156 patients were randomized to receive galunisertib or placebo in the phase II part. Median OS was 7.1 months in the placebo group and 8.9 months in the galunisertib group (HR 0.79; 95% CI: 0.59–1.09; posterior probability HR <1 = 0.93). The drug was well tolerated with a slight increase of neutropenia and fatigue.

## 9. JAK/STAT Inhibitors

Janus kinase/signal transducer and activator of transcription (JAK/STAT) pathway is involved in the signal transduction of several molecules, including cytokines, interleukins, and growth factors [90]. This pathway is involved in a large number of biologic processes, including embryonic development, stem cell maintenance, hematopoiesis, inflammatory response, mitochondrial functions, and epigenetic modifications of chromatin. There are four JAK family members (JAK1, JAK2, JAK3, and TYK2) and seven STAT family members (STAT 1-4, STAT5A, STAT5B, and STAT6) in humans. The activation of this pathway is regulated by a negative feedback, through suppressor of cytokine signaling proteins, and by other regulatory pathways acting at different levels. JAK/STAT pathway is frequently dysregulated in cancers, mainly due to the upregulation of STAT3 and STAT5A/B, and plays an important role in cell proliferation, apoptosis, metabolic changes, EMT, and in prompting an immunosuppressive response [90,91,92]. Napabucasin, an oral STAT3 inhibitor, demonstrated promising activity in a phase Ib/II trial enrolling 59 metastatic PC patients to receive this agent in association with nab-paclitaxel and gemcitabine [93]. DCR was 78.0%, including 2 complete responses (CR) (3.4%) and 26 partial responses (PR) (44.1%). Median PFS and OS were, respectively, 7.1 and 9.6 months. Among 50 evaluable patients, DCR was 92.0%, ORR was 56%, and no dose-limiting adverse events occurred. Based on these results, a randomized phase III study evaluating efficacy and safety of napabucasin combined with nab-paclitaxel and gemcitabine as a first-line treatment for metastatic PC patients was started. Unfortunately, the trial was discontinued for futility following interim analysis [94,95].

Ruxolitinib, a JAK1/2 inhibitor, was tested in two randomized phase III trials in combination with capecitabine in patients with advanced PC experiencing disease progression following first-line therapy [96]. Again, the study was discontinued for futility after interim analysis. To date, the JAK1 inhibitor itacitinib is under evaluation in combination with pembrolizumab for advanced solid tumors, including PC, following encouraging results of the phase Ib/II trial testing this drug in combination with nab-paclitaxel and gemcitabine [97].

## 10. NTRK Inhibitors

The tropomyosin receptor kinase (TRK) family includes three transmembrane protein receptors (TrkA, TrkB, and TrkC, respectively encoded by NTRK1, NTRK2, and NTRK3 genes), which regulate many aspects of neuronal development and function [98,99]. Chromosomal translocations involving NTRK1/2/3 genes result in constitutive activation and aberrant expression of TRK kinases [100]. NTRK alterations are rarely found in tumors with high prevalence, such as PC, where they occur in less than 1% of patients [7]. Recently, specific targeted therapies for NTRK-fusion positive tumors have emerged. Larotrectinib is a highly-selective oral TRK inhibitor that was tested in three single-arm clinical trials by Drillon et al., enrolling a total of 55 patients with TRK fusion–positive cancers [101]. Results were promising; ORR was 75%, with 71% of ongoing responses and 55% of progression-free patients after 1 year. The only PC patients included achieved PR. Median DOR and PFS were not reached.

Based on these results, in November 2018, the Food and Drug Administration approved larotrectinib for the treatment of adult and pediatric patients with NTRK-fusion positive tumors, which are either without a known acquired resistance mutation, metastatic, or in which surgical resection is likely to result in severe morbidity, and without effective alternative treatments or those that have progressed following treatment [102].

## 11. Targeting Cancer Metabolism

Cancer cells present a great number of metabolism modifications, such as alterations in pH homeostasis with related modifications in ion transport systems, that may represent potential targets for therapy [103]. In PC, mutations in genes driving cell growth, such as KRAS, have been demonstrated to alter metabolic pathways, raising interest in agents targeting key molecules [104].

Devimistat is a selective inhibitor of pyruvate dehydrogenase and α- ketoglutarate dehydrogenase, two key enzymes for the tricarboxylic acid cycle of tumor cells. Tricarboxylic acid represents a source of resistance to DNA damaging agents, such that the administration of devimistat supposedly enhances tumor sensitivity to chemotherapeutic agents, such as platinum derivatives [105]. Supporting this hypothesis, a phase I trial testing the combination of devimistat and modified FOLFIRINOX in metastatic PC patients reported a safe toxicity profile and an ORR of 61%, including 3 CRs [106]. Based on these results, a phase III trial testing modified FOLFIRINOX plus devimistat in patients with metastatic PC is currently ongoing (Table 2).

### Hydroxychloroquine

The interest in hydroxychloroquine as a potential antitumor agent derives from its inhibitory effects on cell autophagy. This mechanism represents a defensive strategy against adverse environmental conditions, including an antitumor role during cancer initiation phases. However, cell autophagy can also support tumor growth in a later phase through the catabolism of intracellular organelles, providing nourishment to cancer proliferation [107]. In pretreated metastatic PC patients, a phase II trial that tested hydroxychloroquine (400 mg or 600 mg, twice daily) as a single agent failed to demonstrate antitumor activity [108]. Hypothesizing a synergic effect with chemotherapy, a phase II trial evaluated the addition of hydroxychloroquine to gemcitabine/nab-paclitaxel as a first line therapy for advanced PC patients [109]. The study failed to demonstrate OS benefit with hydroxychloroquine, but ORR was significantly higher in the experimental group compared with chemotherapy alone (38.2% vs. 21.1%, *p* = 0.047). Recently, a phase II study explored the addition of high-dose hydroxychloroquine to gemcitabine/nab-paclitaxel as preoperatory treatment for resectable PC patients [110]. The study documented a significant improvement of pathologic tumor response in the hydroxychloroquine plus chemotherapy arm compared with the chemotherapy alone arm (*p* = 0.00016). Several trials are ongoing with the aim to evaluate the efficacy of hydroxychloroquine in various combinations in PC (Table 1 and Table 2). Interestingly, some of them are testing its combination with MEK inhibitors in RAS-mutant PC, aiming to overcome autophagy-mediate resistance to MEK inhibition [111].

## 12. Immunotherapy

PC is known to be refractory to immunotherapy, mainly due to an immunosuppressive TME characterized by the lack of high-quality effector intratumoral T cells along with a heterogeneous dense stroma acting as a barrier to effector immune cells infiltration [112].

**Figure 2:**
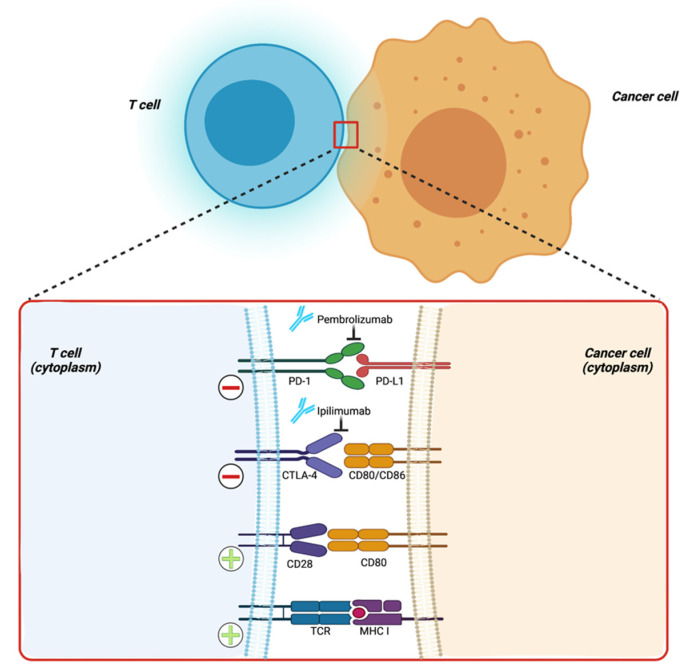
Main immune checkpoints acting on cancer cell recognition by effector immune cells. PD-1: programmed death-1; PD-L1: programmed death ligand 1; CTLA-4: cytotoxic T-lymphocyte antigen 4; TCR: T-cell receptor; MHC I: major histocompatibility complex I; + (plus sign): activating signal; − (minus sign): inhibitory signal. Created with BioRender.com (accessed on 14 July 2021).

The only potential target population of immunotherapy in PC is currently represented by the subset of patients with microsatellite instability high (MSI-H) tumors, in which the immune-checkpoint inhibitor (ICI) pembrolizumab (Figure 2) demonstrated a satisfactory ORR [113,114]. However, this subgroup is only representative of a small proportion (<1%) of PC patients. Efforts to identify a larger population who might benefit from this treatment are therefore remarkably needed.

In a phase II trial by Royal et al., ipilimumab, a fully humanized anti-CTLA-4 IgG1 monoclonal antibody, was administered to 27 patients with locally advanced or metastatic PC, without any survival benefit [115]. A recent phase II trial evaluated a dual immune-checkpoint blockade strategy with anti-CTLA4 and anti-PD-L1 agents, again without encouraging results [116]. A combination strategy using standard chemotherapy (gemcitabine plus nab-paclitaxel) with the addition of the PD-1 inhibitor pembrolizumab was then evaluated in a phase Ib/II trial [117]. Median OS and PFS were 9.1 months and 15.0 months, respectively, but the trial did not meet its primary endpoint of > 15% CR rate. A phase II trial has recently evaluated a dual immune-checkpoint blockade in combination with first-line chemotherapy. A total of 180 patients were randomized 2:1 to receive durvalumab plus tremelimumab in association with gemcitabine plus nab-paclitaxel or chemotherapy alone. Unfortunately, the addition of immune-checkpoint inhibitors did not result in a significant improvement in terms of median OS, PFS, or ORR [118].

Interesting results are emerging from the combination of ICI and vaccines. GVAX, a whole-cell vaccine composed of irradiated and allogeneic PC cells genetically engineered to secrete granulocyte-macrophage colony stimulating factor, seems to convert tumors from non-immunogenic to immunogenic. In addition, its administration results in the upregulation of immune-checkpoint molecules, such as PD-1 and PD-L1, suggesting a potential synergy with ICI [119,120]. In a phase II trial, ipilimumab plus GVAX seemed to induce a benefit in terms of median OS as compared with ipilimumab alone, although not reaching statistical significance [121].

Algenpantucel-L, an allogeneic PC vaccine composed of two human PDAC cell lines (HAPa-1 and HAPa-2) that have been genetically engineered to express αGal by using retroviral transfer of the murine αGT gene, was tested in addition to chemotherapy and chemoradiotherapy as an adjuvant treatment in 70 patients with resected PC [122]. The addition of algenpantucel-L improved both disease-free survival (DFS) and OS (after a median follow-up of 21 months, the 12-month DFS rate was 62%, and the 12-month OS rate was 86%). However, the phase III trial did not confirm the previous findings, showing similar outcomes regardless of the addition of algenpantucel-L to standard treatment [123].

The study of the tumor immune microenvironment is offering new key targets for immunotherapy. Indoleamine 2,3 dioxygenase (IDO) is a tryptophan-catabolizing enzyme that plays a key role in the normal regulation of peripheral immune tolerance as well as in immunotherapy-resistance mechanisms. A phase I study demonstrated promising antitumor activity combining the IDO inhibitor indoximod (1200 mg twice daily) and chemotherapy with gemcitabine/nab-paclitaxel [124]. ORR was 37% (including 1 CR) among 33 patients with metastatic PC.

Inflammatory monocyte recruitment is critical for PC growth and progression. The chemokine (C-C motif) ligand 2 (CCL2)/chemokine (C-C motif) receptor 2 (CCR2) axis drives chemoresistance and immunosuppressive mechanisms in the tumor microenvironment. CCR2 blockade may therefore be a promising immunotherapeutic strategy in PC [125]. PF-04136309, a CCR2 inhibitor, was tested in combination with FOLFIRINOX in patients with borderline-resectable and locally advanced PC, resulting in a 49% ORR [126]. More recently, a phase Ib study tested PF-04136309 in combination with gemcitabine and nab-paclitaxel among 21 patients with previously untreated, advanced PC [127]. However, the antitumor activity was similar to that obtained by chemotherapy alone (ORR 23.8%, and a relatively high (24%) incidence of pulmonary toxicities was observed, raising safety concerns about this combination. Trials currently exploring the efficacy of immunotherapy in PC are summarized in Table 3.

## 13. Conclusions

Therapeutic progress in PC is scant compared to other types of tumors, and chemotherapy is still the mainstay of the treatment. The approval of maintenance olaparib for BRCA-mutant PC has represented an encouraging achievement for personalized medicine in such a recalcitrant disease and opened the doors for the investigation of various agents with potential synergic effects with PARPi, including immunotherapy and tyrosine kinase inhibitors. The emergence of new agents, including tumor-agnostic therapies such as NTRK inhibitors for NTRK-fusion positive tumors or pembrolizumab for MSI-H tumors, warrant the search for novel treatment options in different subsets of PC patients through the implementation of genetic testing. The molecular heterogeneity of PC may demand as much diversified treatment approaches based on individual tumor characteristics.

In conclusion, despite many disappointing results in the past, several investigational therapies have reported promising early outcomes and represent a solid hope for the future of personalized medicine in PC.

## Figures and Tables

**Table 1:** Ongoing trials evaluating activity and efficacy of PARP inhibitors in PC (Clinicaltrials.gov last accessed 10 June 2021).

Target	Tumor	Setting	Treatment Arms	Phase	Primary Outcome	N of Patients	clinicaltrial.gov Identifier
PARP	PC	Advanced, pretreated, without germline BRCA1/2 mutations but with BRCAness phenotype	olaparib	II	ORR	34	NCT02677038
PARP	AST	Advanced, pretreated	(1) AZD6738	II	ORR	68	NCT03682289
ATR			(2) AZD6738 + olaparib				
PARP	AST	Advanced, pretreated	cediranib + olaparib	II	ORR	126	NCT02498613
VEGF							
PARP	PC	Advanced, with BRCA1/2 or PALB2 mutation	(1) veliparib + gemcitabine hydrochloride + cisplatin	II	ORR Dose-finding	107	NCT01585805
			(2) gemcitabine hydrochloride + cisplatin				
			(3) veliparib				
PARP	PC	Metastatic, untreated, with HRD	rucaparib + nal-IRI, leucovorin, fluorouracil	II	ORR	110	NCT03337087
					DLTs		
PARP	AST	Advanced, pretreated, with HRD	rucaparib	II	ORR	220	NCT04171700
PARP	PC	Advanced, pretreated, with BRCA1/2, PALB2, CHEK2 or ATM mutation	niraparib	II	PFS	32	NCT03601923
PARP	PC	Advanced, pretreated	niraparib + dostarlimab + RT	II	DCR	25	NCT04409002
PARP	PC	Advanced, pretreated, with DDR genes alteration	niraparib	II	ORR	18	NCT03553004
PARP	PC	Advanced, following platinum-based CT without PD	(1) niraparib + nivolumab	Ib/II	PFS	84	NCT03404960
			(2) niraparib + ipilimumab				
PARP	PC	Resected, after completion of (neo)adjuvant CT (+/− RT), with BRCA1/2 or PALB2 mutation	(1) olaparib (2) placebo	II	RFS	152	NCT04858334
PARP	PC	metastatic, following platinum-based CT without PD, with BRCA1/2 mutation	(1) olaparib + pembrolizumab (2) olaparib	II	PFS	88	NCT04548752
PD-1							
PARP, PD-1	PC	Metastatic, pretreated, with BRCA1/2, PALB2, BARD1, RAD51c/d mutation	niraparib + dostarlimab	II	DCR	20	NCT04493060
PARP, PD-1	PC	metastatic, untreated, following low-dose CT with gemcitabine, nab-paclitaxel, capecitabine, cisplatin, and irinotecan (GAX-CI)	olaparib + pembrolizumab	II	PFS	38	NCT04753879
PARP	PC	Advanced, untreated, with BRCA1/2 or PALB2 mutation	(1) fluzoparib + mFOLFIRINOX followed by fluzoparib maintenance	Ib/II	DLTs MTD ORR	66	NCT04228601
			(2) placebo + mFOLFIRINOX followed by placebo maintenance				
WEE1	PC	Metastatic, untreated	(1) adavosertib (MK-1775) + nab-paclitaxel + gemcitabine	I/II	MTD PFS	133	NCT02194829
			(2) placebo + nab-paclitaxel + gemcitabine				
RAD51	AST	Advanced, any line	CYT-0851	I/II	DLTs ORR	165	NCT03997968

AST: advanced solid tumor; PC: pancreatic cancer; N: number; PFS: progression-free survival; OS: overall survival; DLTs: dose-limiting toxicities; MTD: maximum-tolerated dose; ORR: objective response rate; DDR: DNA damage response and repair; RT: radiation therapy; RFS: relapse free survival; RP2D: recommended phase 2 dose; HRD: homologous repair deficiency; PARP: poly ADP-ribose polymerase; ATR: ataxia telangiectasia and rad3-related; PD-1: programmed death-1; PD-L1: programmed death ligand 1; VEGF: vascular endothelial growth factor; PD: progressive disease; DCR: disease control rate; mFOLFIRINOX: modified FOLFIRINOX.

**Table 2:** Ongoing trials evaluating activity and efficacy of novel molecules acting on different targets in PC (Clinicaltrials.gov last accessed 10 June 2021).

Target	Tumor	Setting	Treatment Arms	Phase	Primary Outcome	N of Patients	clinicaltrial.gov Identifier
FAK PD-1	PC	Resectable	(1) perioperative CT followed by pembrolizumab + defactinib (2) perioperative CT followed by pembrolizumab	II	pCR rate	36	NCT03727880
FAK	PC	Locally advanced	(1) CT followed by SBRT + defactinib (2) CT followed by SBRT alone	II	PFS	42	NCT04331041
MEK FAK	PC	Advanced, pretreated	GSK2256098 + trametinib	II	ORR	16	NCT02428270
MEK BCL-2	AST	Advanced, pretreated, with KRAS or NRAS mutation	trametinib + navitoclax	Ib/II	ORR PFS Safety	130	NCT02079740
MEK BRAF	PC	Advanced, pretreated, with BRAF V600E mutation	binimetinib + encorafenib	II	ORR	29	NCT04390243
ERK	PC	Metastatic, pretreated	(1) LY3214996 + hydroxychloroquine (2) LY3214996	II	DCR, Safety	52	NCT04386057
ALK5	PC	Metastatic, pretreated	TEW-7197 + FOLFOX	Ib/II	PFS	36	NCT03666832
EGFR	PC	Advanced, pretreated	CT followed by anti-CD3 x anti-EGFR bispecific antibody armed activated T cells	I/II	OS Safety	22	NCT03269526
EGFR HDAC	PC	advanced, first-line	CG200745 PPA + gemcitabine + erlotinib	I/II	ORR	24	NCT02737228
EGFR	PC	Resected, adjuvant	(1) CT (2) gemcitabine + erlotinib	II/III	OS	545	NCT01013649
CTGF	PC	Locally advanced, neoadjuvant	(1) pamrevlumab + gemcitabine + nab-paclitaxel (2) placebo + gemcitabine + nab-paclitaxel	III	OS Proportion of R0 or R1 resection	256	NCT03941093
KRAS	PC	Advanced, pretreated	cyclophosphamide + fludarabine + T cell therapy (+ anti-PD-1)	I/II	ORR Safety	30	NCT04146298
KRAS	AST	Advanced, pretreated, with KRAS G12C mutation	adagrasib	I/II	ORR Safety Plasma concentration	391	NCT03785249
KRAS NRAS	AST	Detectable ctDNA despite prior therapy, with KRAS/NRAS mutation	ELI-002	I/II	MTD RFS Safety	159	NCT04853017
HER2	AST	Advanced, pretreated, with HER2 expression or amplification	A166	I/II	MTD ORR	82	NCT03602079
HDAC PD-1	PC CC	Advanced pretreated	entinostat + nivolumab	II	ORR	44	NCT03250273
XPO1	PC	Metastatic, untreated	(1) selinexor + gemcitabine/nab-paclitaxel gemcitabine/nab-paclitaxel	I/II	MTD OS Safety	56	NCT02178436
NTRK ROS1 ALK	AST	Advanced, with NTRK/ROS1/ALK gene rearrangement	entrectinib	II	ORR	300	NCT02568267
NTRK	AST	Advanced, pretreated, with NTRAK gene rearrangement	larotrectinib	II	ORR	203	NCT02576431
Metabolism	PC	Metastatic, untreated	(1) devimistat + FOLFIRINOX(2) FOLFIRINOX	III	ORR PFS	500	NCT03504423

AST: advanced solid tumor; PC: pancreatic cancer; CC: cholangiocarcinoma; N: number; PFS: progression-free survival; OS: overall survival; DLTs: dose-limiting toxicities; MTD: maximum-tolerated dose; ORR: objective response rate; RT: radiation therapy; RFS: relapse free survival; ctDNA: circulating tumor DNA; FAK: focal adhesion kinase-1; HDAC: histone deacetylase; PD-1: programmed death-1; PD: progressive disease; DCR: disease control rate; pCR: pathologic complete response; XPO1: exportin 1; EGFR: epidermal growth factor receptor; NTRK: neurotrophic tyrosine receptor kinase; ALK: anaplastic lymphoma kinase; ROS1: ROS proto-oncogene 1; KRAS: Kirsten rat sarcoma; NRAS: neuroblastoma RAS viral oncogene homolog; HER-2: human epidermal growth factor receptor 2; ERK: extracellular signal-regulated kinase; BRAF: B-RAF proto-oncogene; Bcl-2: B-cell lymphoma 2; CTGF: connective tissue growth factor.

**Table 3:** Ongoing trials evaluating activity and efficacy of different immunotherapy strategies in PC (Clinicaltrials.gov last accessed 10 June 2021).

Target	Tumor	Setting	Treatment Arms	Phase	Primary Outcome	N of Patients	clinicaltrial.gov Identifier
PD-L1 TGF-βRII	PC	Advanced, pretreated	gemcitabine + nab-paclitaxel + SHR-1701	Ib/II	ORR RP2D	54	NCT04624217
PD-L1 CTLA-4	PC	Locally advanced	minimally invasive surgical microwave ablation + durvalumab + tremelimumab + gemcitabine	II	PFS	20	NCT04156087
PD-1 CTLA-4	PC	Metastatic	nivolumab + ipilimumab + radiation	II	ORR	30	NCT04361162
PD-1	PC	Metastatic	(1) FOLFIRINOX (2) FOLFIRINOX + Anti-PD-1 antibody	III	OS	110	NCT03977272
PD-1	PC	Locally advanced	(1) FOLFIRINOX (2) FOLFIRINOX + anti-PD-1 antibody	III	PFS	830	NCT03983057
PD-1	PC	Metastatic, untreated	gemcitabine + S-1 + nivolumab	II	ORR	38	NCT04377048
CSF1R PD-1	PC	Advanced, pretreated	(1) gemcitabine/nab-paclitaxel or 5-FU/leucovorin/irinotecan liposome (2) cabiralizumab + nivolumab (3) gemcitabine + nab-paclitaxel + cabiralizumab + nivolumab (4) cabiralizumab + nivolumab + FOLFOX	II	PFS	179	NCT03336216
PD-1	PC	Metastatic	(1) FOLFIRINOX/mFOLFIRINOX + anti-PD-1 (2) FOLFIRINOX/mFOLFIRINOX	III	OS	110	NCT03977272
PD-L1 CTLA4	PC	Advanced	(1) 2nd line PD-L1/CTLA4 inhibitor(2) 1st line PD-L1/CTLA4 inhibitor + gemcitabine/nab-paclitaxel (3) 1st line PD-L1/CTLA4 inhibitor + FOLFIRINOX	I/II	ORR	60	NCT04324307
CXCR4 PD-1	PC	Metastatic pretreated	plerixafor + cemiplimab	II	ORR	21	NCT04177810
PD-L1 ICOS	AST	Advanced, pretreated	(1) KY1044 (2) KY1044 + atezolizumab	I/II	ORR Safety	412	NCT03829501
ETBR PD-1	AST	Advanced pretreated	ENB-003 + pembrolizumab	I/II	ORR Safety	130	NCT04205227
PD-1	AST	Advanced, pretreated	(1) pembrolizumab + lenvatinib (2) lenvatinib	II	ORR Safety	760	NCT03797326
CD11b PD-1	AST	Advanced	(1) GB1275 GB1275 + anti PD-1	I/II	ORR Safety	242	NCT04060342

AST: advanced solid tumor; PC: pancreatic cancer; N: number; PFS: progression-free survival; OS: overall survival; ORR: objective response rate; PD-1: programmed death-1; PD-L1: programmed death ligand 1; PD: progressive disease; mFOLFIRINOX: modified FOLFIRINOX; RP2D: recommended phase 2 dose; CTLA-4: cytotoxic T-lymphocyte antigen 4; TGF-βRII: transforming growth factor-beta receptor II; CXCR4: C-X-C motif chemokine receptor 4; CSF1R: colony stimulating factor 1 receptor; ETBR: endothelin B receptor.

## Data Availability

Not applicable.
